# Prevalence and correlates of unhealthy weight control behaviors: findings from the national longitudinal study of adolescent health

**DOI:** 10.1186/2050-2974-2-16

**Published:** 2014-06-03

**Authors:** Eric M Stephen, Jennifer S Rose, Lindsay Kenney, Francine Rosselli-Navarra, Ruth Striegel Weissman

**Affiliations:** 1 Department of Psychology, Wesleyan University, 207 High Street, Middletown, CT 06459, USA; 2 Department of Psychology, Manchester Community College, Great Path MS#4, PO Box 1046, Manchester, CT 06040, USA

**Keywords:** Eating disorders, Purging behavior, Adolescent, Diet pill use, Weight control behavior

## Abstract

**Background:**

A recent study examined the prevalence, clinical correlates, age trends, and stability of unhealthy weight control behaviors (UWCB; purging and diet pill use) in a nationally representative sample of Norwegian boys and girls. The purpose of this study was to provide similar, comparative analyses for a nationally representative sample of American youth.

**Methods:**

Data were extracted from the restricted use data files of survey Waves I, II, and III of the National Longitudinal Study of Adolescent Health (Add Health), selecting all participants who at Wave I had provided information on age, sex, and UWCB. Using UWCB information, three groups were created (purging, diet pill use, and no recent UWCB “controls”) and compared on indicators of adverse health or mental health.

**Results:**

Girls consistently were more likely than boys to report UWCB. UWCB were significantly associated with higher body mass index, self-perception of being overweight, low self-esteem, depression, and delinquency. Prevalence estimates for purging remained relatively constant across the three survey waves; in contrast, diet pill use was especially common at Wave III.

**Conclusions:**

Age trends, gender differences, and clinical correlates of change in the likelihood of UWCB between Waves I-III were all identified in analyses comparing purging and diet pill use in American adolescents. Females and older adolescents were specifically more likely to engage in pill use than purging, and individuals with increased weight dissatisfaction, a history of delinquent behaviors, more depression symptoms, or lower self-esteem were more likely to engage in an unhealthy weight control behavior over time. While the Norwegian study found that prevalence of purging was lower among young adult participants, our results suggested that there were no significant differences in prevalence between age groups.

## Background

Various community surveys have shown that disordered eating behaviors are prevalent in adolescents, correlated with indicators of psychosocial impairment, and predictive of unfavorable physical or mental health outcomes [1-3]. Although disordered eating symptoms such as binge eating, purging, or non-purging compensatory behaviors (for example fasting, excessive exercise, or diet pill use) have often been studied in adolescence as early symptoms of or as proxy variables for full syndrome eating disorders (e.g., anorexia nervosa or bulimia nervosa) [4], investigators have more recently begun to examine disordered eating as a clinically relevant phenomena even in the absence of a diagnostic eating disorder, determined by criteria in the DSM-5 (or DSM-IV).

For example, purging behaviors have received increasing scientific attention as symptoms of clinical relevance in their own right. Studies have shown that vomiting or misusing laxatives or diuretics to manage weight is associated with low self-esteem, depressive symptoms, substance use, and impaired psychosocial adjustment [5-10] and also predict excess weight gain [11]. Results from a large community-based study indicated that women who used extreme weight control behaviors, regardless of whether or not these behaviors occurred in the context of binge eating, reported higher levels of psychopathology and psychosocial impairment [12]. Moreover, several genetic studies have found that, notwithstanding some overlap between latent risk factors for binge eating and purging, there may be distinct risk factors for purging versus binge eating, which warrants further investigation of compensatory behaviors [13,14].

Non-purging forms of weight control behavior have been studied less extensively, yet the limited available evidence indicates that these behaviors also are associated with measures of psychological distress such as anxiety or depressive symptoms [15]. Currently, there is some inconsistency as to whether diet pill use should be classified as a “purging” or “non-purging” weight control behavior [1,15]. Furthermore, research has rarely examined diet pill use outside of these grouped categories, and consequently little is known about the behavior itself.

Two longitudinal studies have reported on changes in purging and non-purging weight control behaviors from adolescence to young adulthood. Project EAT, a longitudinal study of high school students in Minneapolis/St. Paul (United States, US), found an age effect in that, over a 10-year period, the prevalence of diet pill use increased in girls and boys, laxative use increased in girls, and vomiting decreased in girls and boys [1]. New data from Project EAT also suggest a cohort effect: comparisons of two cohorts revealed that fewer students in a cohort recruited in 2010 reported extreme weight control behaviors than in the (original) 1999 sample [16]. The second study (“Young in Norway”) was conducted among a nationally representative sample of Norwegian school children (ages 12 to 20 years at study entry), contrasting purging and non-purging weight control behaviors. In this study, diet pill use was classified as a non-purging behavior. Age differences in rates of purging and non-purging weight control behaviors were examined by grouping participants into four age categories: 14–16, 17–19, 20–22, and ≥ 23 years at each wave. In both boys and girls, prevalence estimates of purging and non-purging weight control behaviors gradually declined with the transition from adolescence to adulthood [15].

The purpose of the present study was to determine whether age trends and predictors of purging and non-purging weight control behaviors observed in Norwegian adolescents [15] could be replicated in a large, nationally representative sample of US youth from the National Longitudinal Study of Adolescent Health (Add Health) where non-purging behavior were limited to diet pill use. First, we tested age trends from adolescence into adulthood in two types of extreme weight control behaviors in boys and girls: vomiting or laxative use as one group, and diet pill use only as a separate group. Consistent with trends observed in the Young in Norway study, we hypothesized decreasing prevalence estimates with increasing age. Second, we examined correlates of these extreme weight control behaviors in boys and girls by comparing them to participants with no recent extreme weight control behaviors. Consistent with previous findings, we hypothesized that more girls than boys would report these extreme weight control behaviors. We further hypothesized that students who reported purging (vomiting or laxative use) or diet pill use for weight control would differ significantly (reflecting greater disturbance) from students who did not engage in any UWCB on measures of body weight, self-esteem, and self-reported depression.

## Methods

### Data source

The present study extracted data from the restricted-use National Longitudinal Study of Adolescent Health (Add Health) survey Waves I (1994–1995), II (1996), and III (2001–2002). Add Health has been following over 20,000 American adolescents over the course of fourteen years, with four waves of data collection since the study’s inception when participants were in grades 7–12. (Wave IV did not collect data about purging and, therefore, was not used for the present study).

Wave I was conducted between 1994 and 1995, with sampling from 132 schools in the United States, representative for region, urbanicity, size, and ethnicity. Initially, 80 high schools were recruited, and each participating school identified a feeder middle school in the community, with the requirement of the inclusion of 7^th^ graders. A total of 20,074 adolescents in grades 7–12 were tested using self-administered, in-school questionnaires given during a 60-minute class period. Wave II was conducted in 1996, and included over 15,000 of the participants originally tested in Wave I. In-home interviews were conducted with participants at Wave III (August of 2001 to April of 2002), with participants now in early adulthood. Further details about the study design can be found elsewhere [17].

Only participants, who provided consistent data across survey waves on birth date and gender, and completed questions concerning vomiting or use of laxatives or diet pills for weight control purposes in Wave I, were included in the present study. A total of 168 participants were dropped due to missing weight control behaviors at Wave I or inconsistent age and gender data across waves.

### Instruments and procedures

Add Health surveys included a wide range of questions pertaining to psychological and physical health issues and behaviors. The variables used in this study are described below.

*Demographic information* was extracted from the Wave I dataset. *Age* was recorded in Waves II and III as age at last birthday. Age at Wave I was calculated by subtracting birth date from the date of interview.

*Unhealthy weight control behaviors (UWCB)*. A two-part question measured whether participants engaged in a number of weight control behaviors. Participants were asked whether, in the past 7 days, they had been trying to lose or maintain weight (yes/no). Participants who answered “no” were instructed to skip the follow-up items; those answering “yes” were asked follow-up questions regarding what specific behaviors were used to do so (response choices included: vomiting; laxative use; diet pill use; dieting; exercising; other; none). Frequency of occurrence was not measured in the Add Health surveys.

*Classification of participants based on unhealthy weight control behaviors*. At each wave, participants were classified into one of three mutually exclusive groups. The *purging group* included those indicating vomiting or using laxatives (regardless of whether they also reported diet pill use) as their means of trying to lose or maintain weight; the *diet pill only group* included those reporting diet pill use but not purging behaviors for weight control purposes. Purging and diet pill use were coded as mutually exclusive in order to analyze diet pill use as a unique behavior and compare pill use with more commonly studied UWCB. Finally, a third group (*no recent UWCB*) comprised all remaining participants, i.e., participants who were not trying to control their weight or participants who reported that they had dieted or exercised (because these behaviors, unless taken to an extreme, are recommended as healthy weight management strategies). Participants reporting “other” behaviors to manage their weight were also classified into the comparison group.

Consistent with the Center for Disease Control (CDC), *Body Mass Index* (BMI) was calculated by dividing weight (in pounds) by the product of height (in inches) squared times the value 703 [18]. *Weight Self-Perception* was measured using a single item (“How do you think of yourself in terms of weight?”). Five responses were available using a Likert scale: “overweight”, “slightly overweight”, “about the right weight”, “slightly underweight”, and “very underweight”.

*Self-esteem* was measured using four questions based on Rosenberg’s Self-Esteem Scale [19] that were asked at each wave (“you have many good qualities”; “you have much to be proud of”; “you like yourself just the way you are”; and “you feel you are doing things just right”). Participants rated each item on a scale from 1 to 5, and a total score was calculated by summing the items [20]. (Cronbach’s alpha =0.79 at Wave I, 0.80 at Wave II, and 0.78 at Wave III).

*Depressive symptoms.* At each wave, participants completed a modified version of the Center for Epidemiologic Studies-Depression (CES-D) scale. Participants rated symptom frequency in the past week on a scale from 0 (never or rarely) to 3 (most of the time). Using the 9 items that were asked at all three waves, we created a total score by adding across all items for each wave. [21] Cronbach’s alpha was 0.81 at Wave I, 0.82 at Wave II, and 0.80 at Wave III. Because of its skewed distribution in our sample, the scale was then natural log transformed.

*Delinquency*. Consistent with other studies that have utilized Add Health data, [22] seven questions concerning delinquent behavior available in all three study waves were used to create a binary delinquency variable. Specifically, participants were asked “In the past 12 months, how often did you: (1) Damage property that didn’t belong to you; (2) Steal something worth more than $50; (3) Go into a house or building to steal something; (4) Use or threaten to use a weapon to get something from someone; (5) sell marijuana or other drugs; (6) steal something worth less than $50; (7) Take part in a fight where a group of your friends was against another group”. Responses to each individual question were dichotomized by recoding the original 4-point Likert format (never, one or two times, three or four times, five times or more) into never versus one or more. The recoded items were then summed. Because the delinquency summary score was extremely skewed with most participants reporting no delinquent behaviors (greater than or equal to 59.0% at all waves), and the majority of the rest reporting just one delinquent behavior (greater than or equal to 20.7% at all waves), we recoded it into a binary categorical variable with a meaningful cut point (0 = no instances, 1 = one or more instances of delinquent behavior).

### Statistical analyses

All analyses were performed using Stata 13.1. Chi-square tests were used first to analyze the differences in prevalence for purging and diet pill use at each wave as well as between boys and girls. Mixed effects logistic regression models were tested to identify changes in these behaviors over the three waves for boys and girls. Mixed effects logistic regression analysis was next used to test for age and gender trends in purging and diet pill use. Finally, mixed effects logistic regression models adjusting for age and gender as covariates were performed to examine differences in the UWCB groups on all measures of psychosocial factors.

## Results

### Sample description

At Wave I, data were available for 10,173 boys and 10,437 girls (mean age = 15.66, SD = 1.75); at Wave II, our sample included 7,108 male and 7,510 female participants (mean age = 16.22, SD = 1.64); and at Wave III, 7,104 male participants and 7,927 female participants (mean age = 21.96, SD = 1.77) were included. In all, 55.99% participants included in our study had data for all three study waves. Efforts to change (gain or lose) weight or maintain weight were reported by a majority of participants. At each wave, about one-third of the participants reported that they were trying to lose weight (34.32%, 33.38%, 33.69% at waves I, II, and III, respectively). Trying to maintain weight also was reported by a substantial subset of participants at Wave I (34.28%) and Wave II (33.11%) but was less common at wave III (15.10%). Fewer participants reported efforts to gain weight than trying to lose or maintain weight at each of the waves (Wave I: 19.66%; Wave II: 18.88%; Wave III: 15.62%). Only a minority of participants at Wave I (11.74%) and Wave II (14.63%) reported that they were not trying to do anything to change or maintain their current weight; at Wave III, about one-third of the sample (35.59%) reported that they made no efforts to change or maintain their weight.

It should also be noted that some attrition bias was found in this sample, such that men were more likely to drop out (OR = 0.90, p < 0.001, CI = 0.85-0.96 at Wave II and OR = 0.71, p < 0.000, CI = 0.67-0.76 at Wave III) as were older participants (OR = 1.56, p < 0.000, CI = 1.53-1.60 at Wave II and OR = 1.09, p < 0.000, CI = 1.08-1.11 at Wave III). However, neither purging nor laxative use at Wave I was associated with attrition at Wave II, and neither purging nor laxative use at Waves I or II was associated with attrition at Wave III.

### Gender and age trends for UWCB prevalence

Table 1 summarizes the prevalence of purging and diet pill use at each wave by gender. As shown in Table 1, significantly more girls than boys reported vomiting, laxative misuse, or using diet pills to lose or maintain weight at each of the three waves. Additionally, because of the small numbers of individuals reporting purging by means of vomiting or laxative misuse, the two groups were combined into one “purging” category, which similarly showed higher rates of purging for girls than boys at all three waves. Mixed effects logistic regression analyses were run to evaluate change in each outcome across the three waves. The results indicated diet pill use increased significantly for men, and both diet pill and laxative use increased significantly for women.

**Table 1:** Prevalence and change in UWCB among male and female participants, Waves I-III

	**Wave I**		**Wave II**		**Wave III**		**Mixed model**		
	**N**	**%**	**N**	**%**	**N**	**%**	**Coefficient**	**Standard error**	**p**
***Males***									
Purging^abc^	24	0.24%	14	0.20%	9	0.13%	0.00	0.00	0.11
Vomiting^abc^	16	0.16%	12	0.17%	4	0.06%	0.00	0.00	0.09
Laxative Use^abc^	8	0.08%	3	0.04%	6	0.08%	0.00	0.00	0.97
Pill Use Only^abc^	17	0.17%	15	0.21%	109	1.54%	0.01	0.00	0.00
No UWCB	10132	99.60%	7077	99.59%	6977	98.34%	–	–	–
***Females***									
Purging	62	0.59%	73	0.97%	57	0.72%	0.00	0.00	0.25
Vomiting	32	0.31%	50	0.67%	23	0.29%	0.00	0.00	0.90
Laxative Use	32	0.31%	29	0.39%	39	0.49%	0.00	0.00	0.048
Pill Use Only	112	1.07%	106	1.41%	409	5.14%	0.02	0.00	0.00
No UWCB	10259	98.33%	7327	97.62%	7489	94.14%	–	–	–

^a^Significant gender differences at T1 (p < 0.05), ^b^Significant gender differences at T2 (p < 0.05), ^c^Significant gender differences at T3 (p < 0.05).

**Figure 1:**
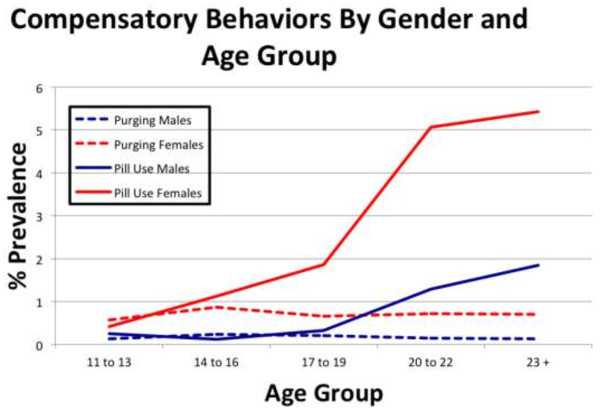
Prevalence for purging and diet pill use from adolescence to young adulthood.

Furthermore, because of the range of ages found in each study wave and the desire to analyze changes both over time and across ages, mixed logistic regression models were also tested with age as a predictor variable, broken into four age categories (14–16, 17–19, 20–22, and 23 or above) to ensure a sufficient sample size in each group. Similar analyses were also performed to test for gender effects overall. Figure 1 illustrates the changes in prevalence of purging and diet pill use from adolescence into early adulthood, and Table 2 shows these age group and gender effects for both the purging (vomiting or laxative misuse) and diet pill only groups against the current UWCB group. As shown in this table, significant gender differences were found for both purging and diet pill use. However, age effects were found only for diet pill use; there was no significant difference for purging across age groups. Further, Table 3 breaks down the prevalence of purging and pill use in each age group by wave, given the possibility for individuals to be classified in the same age group over multiple waves. Age-adjusted prevalence rates were also calculated using population data from the 2000 United States Census due to the higher number of observations in older age groups [23]. In multivariate modeling, however, these repeat observations are also accounted for through the use of mixed models.

**Table 2:** Mixed logistic regression model results for age and gender differences in UWCB groups

	**Purging**	**Pill use only**
	**Coefficient (95% CI)**	**Coefficient (95% CI)**
Age	0.94 (0.83-1.06)	2.01 (1.87-2.16)*
Gender	4.20 (2.95-5.98)*	4.40 (3.62-5.35)*

* - p < 0.001.

**Table 3:** Overall and age-adjusted prevalence of purging and pill use in each age group by Wave

	**Purging**					**Diet pill use**			
**Age group**	**Wave**	**Cases (n)**	**Population (N)**	**Rate per 100,000**	**Age-adjusted rate per 100,000**	**Cases (n)**	**Population (N)**	**Rate per 100,000**	**Age-adjusted rate per 100,000**
11-13	1	13	2846	456.78	89.56	9	2846	316.23	62.01
	2	0	675	0.00	0.00	3	675	444.44	87.15
	3	–	–	–	–	–	–	–	–
	**Total:**	**13**	**3521**	**369.21**	**72.39**	**12**	**3521**	**340.81**	**66.83**
14-16	1	50	10460	478.01	92.52	59	10460	564.05	109.18
	2	50	7259	688.80	133.33	54	7259	743.90	143.99
	3	–	–	–	–	–	–	–	–
	**Total:**	**100**	**17719**	**564.37**	**109.24**	**113**	**17719**	**637.73**	**123.44**
17-19	1	22	7223	304.58	60.33	61	7223	844.52	167.28
	2	35	6502	538.30	106.62	61	6502	938.17	185.82
	3	9	1568	573.98	113.69	45	1568	2869.90	568.44
	**Total:**	**66**	**15293**	**431.57**	**85.48**	**167**	**15293**	**1092.00**	**216.29**
20-22	1	1	77	1298.70	243.20	0	77	0.00	0.00
	2	2	176	1136.36	212.80	3	176	1704.55	319.20
	3	31	7266	426.64	79.90	246	7266	3385.63	634.01
	**Total:**	**34**	**7519**	**452.19**	**84.68**	**249**	**7519**	**3311.61**	**620.15**
23+	1	–	–	–	–	–	–	–	–
	2	–	–	–	–	–	–	–	–
	3	26	6216	418.28	94.12	277	6216	4456.24	1002.77
	**Total:**	**26**	**6216**	**418.28**	**94.12**	**277**	**6216**	**4456.24**	**1002.77**

Those cells where there are no observations are indicated by --. Data used for the weighted rates comes from the 2000 United States Census.

### Clinical correlates for UWCB

Mixed effects logistic regression analysis was then used to examine a range of clinical correlates separately for purging and diet pill use. In each model, age and gender were used as control variables, and each variable of interest was interacted with time in order to ascertain the main effect of the variable as well as its effect over time. Because of the high correlation between perceived weight image and BMI, these variables and their interaction with time were tested together in a model to determine whether they were independently associated with purging and pill use. Results are shown in Table 4.

**Table 4:** Mixed logistic regression results for differences in clinical correlates for each UWCB group

	**Purging**				**Pill use**			
	**Coefficient**	**SE**	**p**	**95% CI**	**Coefficient**	**SE**	**p**	**95% CI**
Females dompared to males	1.21	0.17	0.00	0.87-1.55	1.51	0.10	0.00	1.30-1.71
Age difference	−0.06	0.03	0.07	−0.12-0.01	0.15	0.02	0.00	0.11-0.19
CESD (log trans) Difference	0.72	0.16	0.00	0.41-1.03	0.42	0.10	0.00	0.23-0.63
Linear change across waves	0.12	0.28	0.67	−0.42-0.66	0.76	0.14	0.00	0.23-0.63
CESD Difference over time	0.11	0.12	0.39	−0.14-0.35	−0.13	0.06	0.02	−0.24-0.02
Females compared to males	1.26	0.18	0.00	0.92-1.61	1.47	0.10	0.00	1.27-1.67
Age difference	−0.07	0.03	0.05	−0.13-0.00	0.14	0.02	0.00	0.10-0.19
Self-esteem difference	−0.16	0.04	0.00	−0.23- -0.09	−0.24	0.03	0.00	−0.30- -0.19
Linear change across waves	1.10	0.46	0.02	0.20-2.00	−0.32	0.28	0.26	−0.87-0.23
Self-esteem difference over time	−0.05	0.03	0.08	−0.11-0.01	0.06	0.02	0.00	0.03-0.09
Females compared to males	1.51	0.18	0.00	1.16-1.86	1.68	0.11	0.00	0.12-0.20
Age difference	−0.04	0.03	0.20	−0.11-0.02	0.16	0.02	0.00	0.12-0.20
Delinquency difference	0.63	0.21	0.00	−0.22-1.04	0.66	0.17	0.00	0.32-1.00
Linear change across waves	0.21	0.15	0.17	−0.09-0.51	0.55	0.10	0.00	0.35-0.76
Delinquency difference over time	0.07	0.18	0.70	−0.28-0.41	−0.06	0.11	0.59	−0.28-0.16
Females compared to males	1.13	0.18	0.00	0.77-1.49	1.25	0.11	0.00	1.03-1.46
Age difference	−0.08	0.04	0.02	−0.15- -0.01	0.11	0.02	0.00	0.07-0.15
Weight image difference	0.93	0.18	0.00	0.59-1.28	0.99	0.15	0.00	0.70-1.28
Linear change across waves	1.05	0.47	0.03	0.13-1.96	0.42	0.30	0.17	−0.18-1.01
Weight image difference over time	−0.02	0.15	0.88	−0.31-2.66	0.06	0.09	0.54	−0.12-0.28
BMI Difference	0.03	0.02	0.14	−0.01-0.08	0.04	0.02	0.06	0.00-0.07
BMI Difference over time	−0.03	0.02	0.09	−0.06-0.00	−0.01	0.01	0.52	−0.03-0.01

Note: CESD = Center forEpidemiologic Studies Depression scale; SE = standard error; p = significance level; CI = confidence interval.

Significant overall group differences were found for each of the clinical correlates examined in this study, with the exception of BMI. Participants who either purged or used diet pills as a form of UWCB perceived themselves as more overweight, reported higher depression symptom scores, reported lower self-esteem, and were more likely to have engaged in delinquent behaviors. After adjusting for weight image, BMI was not significantly associated with either the purging or diet pill group. Significant interactions with time were found in diet pill use for depression scores and self-esteem. Participants with higher levels of depression and lower self-esteem at Wave I showed a smaller increase in diet pill use over time. None of the clinical correlates were associated with changes in the likelihood of purging over time.

## Discussion

This study examined prevalence and correlates of UWCB as well as trends over time in these behaviors in a large, nationally representative sample of males and females. Consistent with prior research, UWCB were reported by far more female participants than male participants [1,15]. The prevalence estimates obtained in our sample for vomiting or laxative misuse for weight control reasons are lower than estimates reported in other studies [15], most likely due to the short time frame used in Add Health for measuring UWCB (past 7 days). Regardless of how many adolescents engage in purging, experts uniformly agree that vomiting or laxative abuse is a health risk behavior and should be addressed via therapeutic or preventive interventions [15].

Individuals who acknowledged purging had greater weight dissatisfaction than those in the no recent UWCB group. These results are noteworthy because the no recent UWCB group included participants who reported using various behaviors to try and lose or maintain weight that were not considered to be UWCB as defined in this study. This makes our comparisons more conservative than had we used a comparison group comprised of individuals who did not report any efforts to manage their weight.

Prior studies have shown that UWCB actually are associated concurrently and prospectively with increased weight [1]. Moreover, also in keeping with prior studies, compared to the no recent UWCB group, the purging group scored lower on self-esteem and higher on depression [4]. At Wave I, the purging group was more likely than the no recent UWCB group to report having engaged in delinquent behaviors; however, there was no significant difference in the likelihood of purging or diet pill use over time for those engaged in delinquent behavior compared to those who did not. Both purging and delinquent behaviors fall in the spectrum of disinhibited or impulsive behavior. Reports of delinquent behavior declined with each successive wave in all three groups, possibly reflecting increasing maturity and self-control, although rates of purging showed no evidence of declining over time. Additional studies are needed to further explore the relationship between delinquent behavior and purging.

It is important to note that the models testing both BMI and weight dissatisfaction showed significant associations between weight dissatisfaction for both UWCB outcomes, but there were no significant associations for BMI. This may be due to the fact that a significant number of individuals in this sample who did not show any recent UWCB were also trying to lose or maintain weight. Future research may wish to investigate how these purging and pill use groups differ specifically from those who were not trying to lose or maintain weight as opposed to the no recent UWCB group more broadly in order to more fully understand the differences between weight dissatisfaction and BMI as clinical correlates.

Overall, our findings suggest that purging and diet pill use are associated with a less favorable profile on the clinical correlates examined in this study. Among those who reported purging or diet pill use only to manage their weight, these correlates suggest more pathology compared to the no recent UWCB group, including higher weight dissatisfaction, greater prevalence of delinquent behaviors, lower self-esteem, and increased depression. However, only depression and self-esteem were associated with change in the likelihood of pill use over time, and none of the clinical correlates were related to changes in the likelihood of purging. Research is needed to determine whether educating individuals about the lack of efficacy of purging as a long-term weight management strategy would reduce the prevalence or incidence of these behaviors.

To our knowledge, ours is the first study to examine the prevalence of diet pill use in the absence of purging behaviors [24]. Diet pill use was considerably more prevalent than purging, and unlike purging, showed a considerable increase with age. At Wave I, diet pill users were significantly older than purgers. More importantly, the diet pill group differed significantly from the no recent UWCB group on all clinical correlates with the exception of BMI. Diet pill use was associated with greater weight dissatisfaction, more delinquent behaviors, and less favorable scores on measures of psychological adjustment such as self-reported depression or self-esteem at Wave I. In addition, higher levels of depression and lower self-esteem were associated with an increased likelihood of diet pill use over time.

In contrast to findings from a Norwegian study where the prevalence of purging was found to be lower among late adolescent or young adult participants [15], our findings from the Add Health sample suggest that purging prevalence remained relatively unchanged across the age groups, and diet pill use was far more prevalent among participants in the age groups of 17 years and older. Thus, the development of these behaviors may be different for American youth, and more research on predictors of purging—particularly diet pill use in the absence of purging—is warranted to further identify how these behaviors develop in American youth.

Several limitations need to be acknowledged. The Add Health measures used in our analyses relied on self-report, which is subject to recall errors or biased reporting. The time frame used for measuring presence of UWCB was shorter (past 7 days) than is typical in most epidemiological studies of disordered eating. Therefore, it is unclear the extent to which individuals in the comparison group (no recent UWCB) may have also engaged in diet pill use, laxative misuse, or vomiting for weight control purposes during the longer time frame covered in some of the psychosocial measures. For example, no time frame was specified for measuring self-esteem, and delinquency was based on the past 12 months. Inadvertent inclusion in the no recent UWCB group of individuals who might have reported “yes” to the question about purging or diet pill use had the time frame been broader renders our group comparisons more conservative. Similarly, those who reported “other” mechanisms with which to lose or maintain weight were not asked about what those mechanisms were, and therefore it is possible that these could be classified as UWCB.

Limitations are also associated with several of the variables used in this study. For example, Add Health did not collect information on the type of diet pills used. Similarly, data on binge eating, a symptom often associated with purging or diet pill use, were not collected. In addition, because frequency information was not available for UWCB, we were unable to study the severity of these behaviors. Finally, low frequency of engagement in delinquent behaviors limited us to defining delinquency more broadly as a binary yes/no for engagement in delinquent behaviors and precluded an examination of each behavior separately.

Another potential limitation arises from our classification of purging and diet pill use. Specifically, despite the fact that this was a large, nationally representative, epidemiological study, the low prevalence of UWCB required that we group individuals who vomited and those who abused laxatives into one group. Larger samples are needed to determine whether these two types of purging behavior should be classified into distinct groups. This may require oversampling on these behaviors given their low prevalence rate in the population. Moreover, we prioritized purging over diet pill use, allowing into our purging group individuals who also used diet pills but not vice versa (i.e., the diet pill group only reported diet pill use and not purging).

Finally, concerns arise regarding the analyses in Tables 2 and 3 given that not every subject has an equal probability of contributing to the various age-specific prevalences. While the use of the mixed modeling helps to deal with this issue, such unequal opportunities may present a problem. This concern is also compounded by the fact that men and individuals who were older were significantly more likely to drop out of the study at a follow up point, and thus the results of this study must be interpreted with caution.

Nonetheless, these limitations were offset by several strengths. The study involved a nationally representative sample, and therefore did not contain the limitations inherent in studies of patient samples. Furthermore, the large sample size afforded us the opportunity to analyze both men and women, and the longitudinal design allowed us to gain information on our sample over a considerable period of time (8 years) from adolescence into early adulthood. Finally, the classification system allowed an opportunity to study diet pill use, a previously understudied behavior, as a unique UWCB.

## Conclusion

This study examined the prevalence of vomiting, laxative misuse, and diet pill use among adolescents, as well as the age trends of these behaviors from adolescence into adulthood and the clinical correlates that predict diet pill use and purging UWCB. Results suggest that girls or women are more likely than boys or men to engage in each of these behaviors and that diet pill use, but not vomiting or laxative misuse, increases over time. Furthermore, results have shown significant differences between diet pill use, purging UWCB, and the control group for a variety of clinical correlates, supporting research indicating that it is important to prevent, identify, and treat nonclinical levels of disordered eating. Our results differed from the Norwegian study in that we did not find that purging prevalence was lower in young adults. Whether these differences between our and the Norwegian study reflect cultural differences or are a function of methodological differences awaits further investigation.

## Competing interests

The authors have no conflicts of interest to report.

## Authors’ contributions

EMS conducted all statistical analyses, contributed to Methods selection, provided data interpretation, and wrote the Results and Discussion sections of the manuscript. JR contributed to interpretation of data and revisions to drafts of the manuscript. LK contributed to the conception of the study as well as the drafting of introduction and discussion sections of the manuscript. FR made significant revisions to the original manuscript for intellectual content. RS made substantial contributions to the original manuscript and supervised all data analysis and interpretation. All authors read and approved the final manuscript.
